# Comparison of erythrocyte sedimentation rate measurement between Westergren method and automated method among patients attending Jigjiga University Sheik Hassen Yabare Referral Hospital, Jigjiga, Ethiopia

**DOI:** 10.3389/fmed.2024.1414097

**Published:** 2024-08-01

**Authors:** Muluken Walle, Ermiyas Alemayehu, Addisu Tesfaye, Mesay Arkew, Haftu Asmerom, Melaku Mekonnen Agidew, Fasil Getu

**Affiliations:** ^1^Department of Medical Laboratory Sciences, College of Medicine and Health Sciences, Jigjiga University, Jigjiga, Ethiopia; ^2^Department of Medical Laboratory Sciences, College of Medicine and Health Sciences, Wollo University, Dessie, Ethiopia; ^3^School of Medical Laboratory Sciences, College of Health and Medical Sciences, Haramaya University, Harar, Ethiopia; ^4^Department of Medical Biochemistry, College of Health Sciences, Debre Tabor University, Debre Tabor, Ethiopia

**Keywords:** erythrocyte sedimentation rate (ESR), Westergren, automated, SFRI, Jigjiga, Ethiopia

## Abstract

**Introduction:**

Erythrocyte sedimentation rate (ESR) is a widely used screening test in clinical practice as an indicator of inflammatory and degenerative malignant diseases. The Westergren method, renowned as the gold standard, is valued for its accuracy and cost-effectiveness but demands considerable time and blood volume. Emerging automated methods offer quicker and more convenient alternatives, aiming to replace manual techniques. Nonetheless, validating these automated methods against the reference Westergren method is essential to ensure reliability. Therefore, this study aimed to evaluate ESR measurement results obtained from both the reference Westergren method and the automated (SFRI ESR 3000) method.

**Methods:**

A Hospital-based comparative cross-sectional study was conducted at Jigjiga University Sheik Hassen Yabare Referral Hospital from July 15 to September 16, 2023. Following the acquisition of informed consent, blood samples were obtained from 158 participants, five milliliters of blood from each participant. These samples were then subjected to ESR estimation using both the Westergren (reference) method and the automated (SFRI ESR 3000) method. Subsequently, the collected data were analyzed using SPSS version 20 and MedCalc version 12.3.0.0 statistical Softwares. Statistical analyses such as Paired *t*-tests, Pearson correlation, linear regression, and the Bland and Altman plot were employed. A *p*-value of < 0.05 was considered statistically significant.

**Results:**

The paired sample *t*-test analysis revealed no significant difference between the use of the reference Westergren method and the automated method for ESR determination, with a mean difference (MD) of 0.7 ± 9.2 mm/h (*P* = 0.36). Additionally, a significant correlation was observed between the two methods, with a remarkable correlation coefficient (*r* = 0.94, *p* < 0.001). The Bland–Altman data analysis indicated no evidence of systematic bias and demonstrated good agreement of ESR values between the two methods, with a limit of agreement of −17.3 to +18.7. Moreover, within-run imprecision analysis for the automated method across a range of ESR values showed coefficient of variation of 27.08, 12.65, and 10.32% for low, medium, and high ESR levels, respectively.

**Conclusions:**

The SFRI ESR 300 automated method demonstrates the potential for interchangeable use with the Westergren method for determining ESR, given the strong correlation and good agreement. Additionally, the same reference range could be applied during interpretation.

## Introduction

The erythrocyte sedimentation rate (ESR) is a common hematology test indicating the rate of red blood cell (RBC) sedimentation when anti-coagulated blood in a standardized tube is allowed to stand undisturbed for a specified period. This process occurs in three distinct phases: aggregation, precipitation, and packing (1). ESR evaluation is frequently used to assess the acute phase response in pathological conditions, suggesting the presence of inflammation, infection, trauma, or malignant disease (2). Various physiological and pathological factors, such as hemoglobin concentration, ratio of RBCs to plasma proteins, serum lipid concentration, and plasma PH, can influence ESR values (3). Additionally, RBC concentration, anisocytosis, and poikilocytosis have an impact on ESR values (4).

Erythrocyte sedimentation rate can be measured using different methods with varying methodologies and principles, including the Westergren method and automated analyzers (5). Clinical laboratories prioritize factors such as the safety of laboratory personnel, ease of operation, and reduced turnaround time (6). The Westergren method, commonly employed and endorsed as the gold standard by the International Committee for Standardization in Hematology (ICSH), involves placing anti-coagulated blood in an upright tube (Westergren tube) and measuring the distance RBCs have fallen in millimeters after 1 h (1, 7). Despite its simplicity and cost-effectiveness, this method is time-consuming and requires a relatively large blood volume (1).

Several newer automated analyzers for measuring ESR have been developed and introduced in clinical laboratories to address many of the concerns encountered previously (8). Most automated analyzers do not directly measure sedimentation; instead, they calculate a mathematically derived rate based on aggregate measurements in the early stages of RBC clumping, known as rouleaux formation (5). These newer automated methods offer numerous advantages, including operator safety, reduced biohazard risks, and shortened measurement times (8).

In recent years, closed automated systems capable of measuring ESR directly from a capped ethylene diamine tetraacetic acid (EDTA) blood sample tube have been developed (6). The use of undiluted EDTA blood increases sample stability and allows for the use of a single sample for both ESR measurement and other hematologic tests (9). Furthermore, this method minimizes the possibility of external influences such as temperature, dust particles, tube positioning, and diluent ratios affecting the final reading (10).

Many new automated systems have been introduced and evaluated for their performance against the gold standard Westergren method (11). Most studies indicate that ESR measurements using automated methods have high comparability with the Westergren method (12). However, some studies have found differences in ESR results obtained with the new instruments compared to those obtained with the Westergren method (13). It is important to note that all ESR methods should align with the ICSH recommended method (7), underscoring the need for manufacturers and healthcare facilities to validate and verify new methods (5). Therefore, this study aimed to evaluate ESR measurement results between the reference Westergren method and the automated method.

## Methods

### Study area, design, and period

A hospital-based cross-sectional study was conducted at Jigjiga University Sheik Hassen Yabare Referral Hospital (JUSHYRH) from July 15 to September 16, 2023. The hospital is situated in Jigjiga town, which is located ~675 km east of Addis Ababa, the capital of Ethiopia.

### Study population

The source population for this study comprised all patients who attended the outpatient department (OPD) of JUSHYRH. The study population included all patients of both genders and all age groups who visited the OPD during the study period and had been requested to undergo an ESR test based on the treating physician's order.

### Sample size determination and sampling technique

Based on the rule that has been recommended by the Clinical and Laboratory Standards Institute (CLSI), a minimum of 100 patient samples are required to detect real differences and establish bias claims between two measurement procedures (14). Accordingly, a total of 158 patients who had been requested for ESR tests from the OPD ward were recruited. Thus, ESR measurements using both Westergren and automated methods were done for each collected sample. The study participants were selected using a systematic random sampling technique. In JUSHYRH, the estimated number of requested ESR tests from the OPD ward for three data collection months was 885, obtained from registered logbooks. The “K” interval for selecting specific study participants was calculated by dividing the estimated value by our sample size: K = N/n = 885/158 = 6. Therefore, the first patient (the first sampling unit) was randomly selected between 1 and 6 and the next participant was selected every 6th interval until the calculated sample size (158) was settled.

### Data collection

After obtaining informed written consent, socio-demographic and clinical data of each participant, including age, gender, marital status, educational status, and occupation status, were collected using a structured questionnaire through face-to-face interviews.

### Laboratory sample collection and ESR determination

Erythrocyte sedimentation rate measurements were performed for all participants using both the reference (Westergren) and automated (SFRI ESR 3000) methods. Accordingly, 5 ml of venous blood specimen was collected from each study participant using a syringe and needle method aseptically by laboratory professionals. Then, 1.6 ml of whole blood was gently mixed with 0.4 ml of 3.8% sodium citrate for measurements of ESR by Westergren method. On the other hand, 3 ml of whole blood was transferred into K2-EDTA vacuum tubes for ESR measurements by automated analyzer. The manual Westergren method was applied by diluting four volumes of blood with one volume of sodium citrate, following the ICSH protocol (7). The diluted anti-coagulated blood was aspirated into a 200 mm glass Westergren pipette (Vacuette, Greiner bio-one) and placed in a vertical stand. The sedimentation rate of the RBCs was visually recorded by measuring the column of plasma from the top of the pipette to the upper limit of RBC sedimentation after 1 h. The automated ESR was measured using the SFRI ESR 3000 (SFRI Medical Diagnostics, France), an automatic ESR analyzer capable of performing standardized ESR analysis compliant with the modified Westergren method. This analyzer operates on the principle of photometric infrared reading and can process 30 samples simultaneously with random access. Results were categorized into three groups: low, medium, and high ESR levels based on their sedimentation range.

### Data quality assurance and management

To ensure consistency and accuracy of ESR measurements for both Westergren and automated methods, manufacturer instruction and standard operating procedure of each method were strictly followed, including sample handling, mixing, and measurement protocols. Reference control materials with known ESR values were used to calibrate the instruments. Factors that potentially affect ESR result such as temperature, sample volume, and instrument sensitivity were regularly controlled and adjusted to ensure accurate readings. Furthermore, each specimen was checked for any hemolysis and clotting before testing. All blood samples were analyzed within 2 h of specimen collection.

### Statistical analysis

EpiInfo version 3.5.4 was utilized for data entry, while SPSS version 20 and MedCalc version. 12.3.0.0 were employed for statistical analysis. Data distribution was checked by the Shapiro-Wilk normality test. Statistical analysis included Paired *t*-tests at a 95% confidence interval (CI) to compare ESR values between manual and automated methods. Pearson correlation coefficient (*r*) was calculated to evaluate the correlation between the two methods Linear regression analysis was performed according to Passing–Bablok. The Bland and Altman plot was used to assess bias and limits of agreement (LoA) between the two methods. In this method, differences in ESR values between the two methods were plotted against mean values. Agreement was deemed acceptable when the difference fell within mean ± 2 SD (mean ± 1.96 SD) for a 95% CI (15). A *p*-value of < 0.05 was considered statistically significant. Additionally, the imprecision was expressed as the within-run coefficient of variation (CV) using replicate measurements within different category of ESR levels.

## Results

### Demographic characteristics of study participants

A total of 158 study participants who had been requested for ESR test were included in the study. The majority of participants were female (53.8%), resided in rural areas (55.1%), and were married (60.1%). In terms of educational status, the majority of participants had completed primary school (31.6%), followed by secondary school (28.5%). The participants' ages ranged from 14 to 84 years, with a mean ± SD age of 39.7 ± 16.8 years. Furthermore, the mean ± SD age for men was 41 ± 2.1 years, with an age range of 14–81 years, while for women, it was 37.9 ± 16.5 years, with an age range of 14–84 years (Table 1).

**Table 1:** Socio-demographic characteristics of study participants for the comparison of Westergren method and automated (SFRI ESR 3000) method at JUSHYRH.

**Variable**	**Category**	***N***	**ESR measurement methods**		**Mean difference (mean ±SD)**	***P*-value**
			**Westergren (mean ±SD)**	**Automated (mean ±SD)**		
Gender	Male	73	38.8 ± 25.7	37.5 ± 24.9	1.3 ± 8.7	0.21
	Female	85	52.1 ± 26.5	51.9 ± 27.9	0.1 ± 9.6	0.91
Age	< 25 years	37	44.1 ± 23.9	42.9 ± 24.8	1.1 ± 8.5	0.44
	25–50 years	82	45.9 ± 28.7	44.8 ± 29.2	0.9 ± 10.1	0.41
	>50 years	39	48.2 ± 26.1	48.5 ± 26.4	−0.31 ± 7.8	0.81
Residence	Rural	87	42.9 ± 26.8	42.3 ± 27.2	0.6 ± 8.9	0.51
	Town	71	49.5 ± 26.7	48.8 ± 27.6	0.7 ± 9.6	0.54
Educational status	Unable to read and write	30	43.2 ± 29.0	43.6 ± 30.1	−0.4 ± 9.5	0.82
	Primary	50	46.9 ± 26.7	46.3 ± 27.9	0.6 ± 8.9	0.65
	Secondary	45	47.8 ± 24.5	45.8 ± 23.8	1.9 ± 10.3	0.22
	Above degree	33	44.3 ± 29.2	44.3 ± 29.9	0.0 ± 7.7	0.98
Marital status	Married	95	43.9 ± 28.1	42.8 ± 27.1	1.1 ± 8.9	0.25
	Single	44	45.1 ± 24.3	43.9 ± 25.8	1.1 ± 8.6	0.39
	Divorced	12	57.3 ± 29.5	59.4 ± 35.9	−2.2 ± 12.3	0.55
	Widowed	7	59.4 ± 13.9	62.4 ± 14.3	−3.0 ± 10.3	0.47

N, number of participants; ESR, Erythrocyte sedimentation rate; SD, standard deviation; CI, confidence interval.

### Comparison of ESR values using automated with manual Westergren method

The ESR measured using the reference Westergren method ranged from 6 to 120 mm/h, while the ESR measured by the automated method (SFRI ESR 3000) ranged from 5 to 120 mm/h. Since the distributions of the data were normal Comparison between the two measurement methods was done by *t*-test, and the results are expressed as mean ± SD. The mean ± SD values of ESR were 45.9 ± 26. 9 mm/h and 45. 3 ± 27.4 mm/h using the Westergren method and the automated method, respectively. The paired sample *t*-test analysis revealed that there was no significant difference between the use of the manual reference Westergren method and the automated method for ESR determination, with a mean difference (MD) of 0.7 7 ± 9.2 mm/h (*P* = 0.36) and a *t*-value of 0.9 at 157 degrees of freedom and a 95% level of significance (Table 2).

**Table 2:** Paired samples *t*-test of the ESR values using manual Westergren method and automated (SFRI ESR 3000) method among the study participants at JUSHYRH.

**ESR measurement (mm/h)**		***N***	**ESR measurement methods**		**Paired mean difference (mean ±SD)**	***t*-value**	**Df**	***P*-value**
			**Westergren (mean ±SD)**	**Automated (mean ±SD)**				
All ESR measurements		158	45.9 ± 26.9	45.3 ± 27.44	0.7 ± 9.2	0.9	157	0.36
Categorized ESR measurement based on range	Low (0–20 mm/h)	34	12.7 ± 4.3	13.9 ± 6.1	−1.3 ± 5.0	−1.5	33	0.14
	Medium (21–80 mm/h)	104	47.7 ± 16.3	46.9 ± 19.4	0.8 ± 9.8	0.9	103	0.39
	High (>80 mm/h)	20	93.3 ± 11.7	90.1 ± 15.04	3.2 ± 11.1	1.3	19	0.21

N, number of participants; ESR, Erythrocyte sedimentation rate; SD, standard deviation; t, T-test; df, degree of freedom; CI, confidence interval.

**Figure 1:**
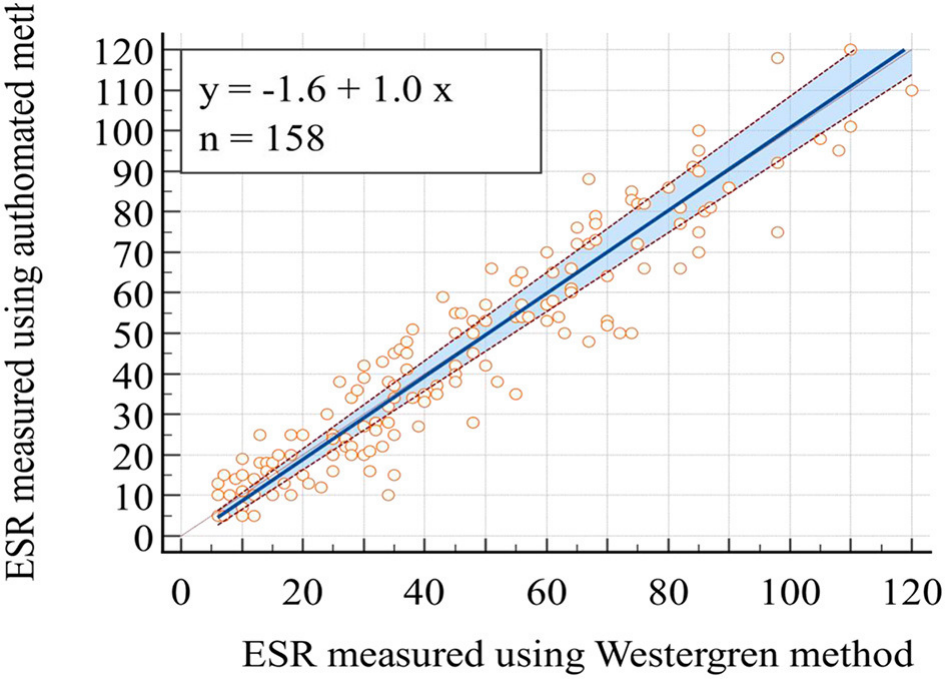
Plot of Passing Bablok regression analysis for the comparison of ESR values obtained through the reference Westergren method and the automated (SFRI ESR 3000) method.

Pearson's correlation coefficient and least square linear regression analysis were employed to assess the association between the two ESR measurement methods. The correlation analysis revealed a significant correlation between the two methods, with a remarkable correlation coefficient of *r* = 0.94 (95% CI; 0.92–0.96), *p* < 0.01 (Table 3). The Passing Bablok regression analysis yielded the equation “y = 1.0x −1.6” between the two methods. This equation predicts ESR values for the automated method from ESR values of the reference method: Automated ESR values = 1.0 × Westergren ESR values −1.6 The results of the intercept CI were obtained at −1.6 (95% CI: −3.2 to −0.2) and slope of 1.0 (95% CI: 0.9 to 1.1; Figure 1).

**Table 3:** Evaluation of 95% limits of agreement, and correlation coefficient of ESR measurements using the reference Westergren method and automated (SFIR ESR 3000) method.

**ESR measurement**		**N**	**ESR measurement methods**	**Range (mm/h)**	**Mean ±SD (mm/h)**	**Mean difference (95% LoA)**	**Pearson's correlation coefficient (*r*)**
All ESR measurements		158	Westergren	6–120	45.9 ± 26.9	0.7	0.94
			Automated	5–120	45.3 ± 27.4	(−17.3, 18.7)	(p < 0.01)
Categorized measurement of ESR based on range	Low	34	Westergren	6–20	12.7 ± 4.3	−1.3	0.59
	(0–20 mm/h)		Automated	5–25	13.9 ± 6.1	(−11.1, 8.5)	(p < 0.01)
	Medium	104	Westergren	21–80	47.7 ± 16.3	0.8	0.87
	(21–80 mm/h)		Automated	10–88	46.9 ± 19.4	(−18.3, 19.9)	(p < 0.01)
	High	20	Westergren	82–120	93.3 ± 11.7	3.2	0.68
	(>80 mm/h)		Automated	66–120	90.1 ± 15.0	(−18.6, 25.0)	(p = 0.01)

N, number of participants; ESR, Erythrocyte sedimentation rate; SD, standard deviation; LoA, limits of agreement; CV, coefficient of variation; r, Pearson's Correlation coefficient.

**Figure 2:**
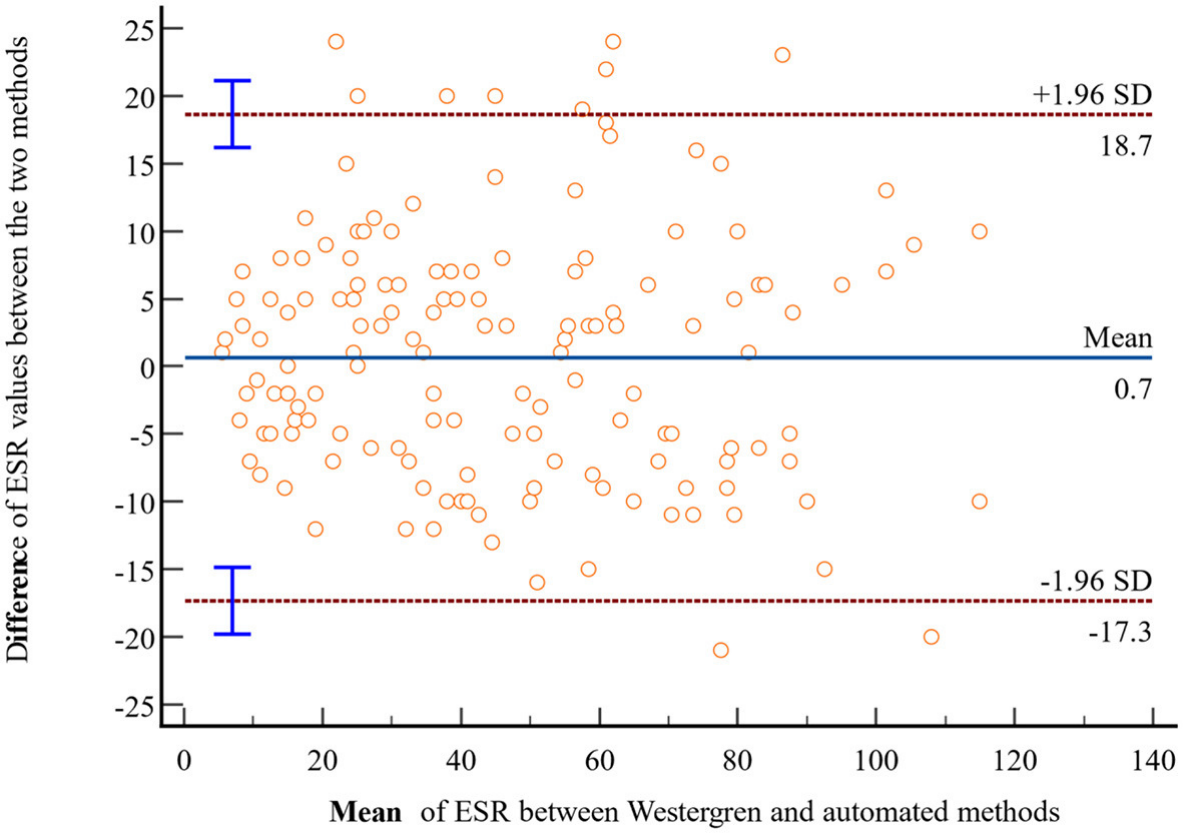
Bland-Altman plot comparing ESR values obtained through the reference Westergren method and the automated (SFRI ESR 3000) method among study participants at JUSHYRH.

In light of the statistically insignificant findings from the *t*-test analysis, a Bland-Altman plot was employed to assess the agreement of ESR values obtained through the Westergren and automated methods. The Bland-Altman analysis is plotted using the difference of ESR values obtained with the Westergren and automated methods against the mean of the two ESR measurements. The result revealed no discernible systematic bias, indicating a strong concordance in ESR values between the Westergren and SFRI ESR 3000 automated methods. Specifically, 95% of the data points fell within a narrow range of LoA, calculated as follows: the lower LoA d-(1.96 × SD) = 0.7– (1.96 × 9.2) = −17.3 and d+(1.96 × SD) = 0.7+(1.96 × 9.2) = +18.7 (Figure 2). Furthermore, to investigate the potential presence of proportional bias, linear regression analysis was conducted using the difference between the two methods as the dependent variable and the mean of the measurements as the independent variable. The results demonstrated no evidence of proportional bias in the Bland-Altman plot, with a non-significant *p*-value of 0.45.

### Evaluation of within-run imprecision

It is imperative to evaluate new technologies across a spectrum of ESR values. The CV% is used to reflect the precision of the measurement method. The within-run CV% was calculated using three patients samples with ESR values of 5, 45, and 90 mm/h which were representing low (ESR < 20 mm/h), medium (ESR 20–80 mm/h), and high (ESR > 80 mm/h) ESR levels, respectively. The samples were categorized into three range groups based on ESR values obtained by the Westergren method. Then, 10 replicate measurements were made on each sample using Westergren method and automated (SFRI ESR 3000) method. Mean, SD, and CV% were computed for each ESR category and method. The results revealed within-run imprecisions of 27.08, 12.65, and 10.32% for the automated method across low, medium, and high ESR levels, respectively. Notably, more variations were observed for low ESR values followed by medium values for the automated ESR determination method (Table 4).

**Table 4:** Evaluation of within-run Imprecision (CV%) at low, medium, and high ESR levels using Westergren and automated (SFRI ESR 3000) methods.

**Categorized ESR measurement**	**ESR measurement methods**	**Number of replicate measurement**	**Mean ±SD (mm/h)**	**CV%**
Low (0–20 mm/h)	Westergren	10	7.00 ± 1.70	24.28%
	Automated	10	9.70 ± 2.63	27.08
Medium (21–80 mm/h)	Westergren	10	47.30 ± 7.10	15.02%
	Automated	10	43.30 ± 5.48	12.65%
High (>80 mm/h)	Westergren	10	94.90 ± 9.66	10.17%
	Automated	10	92.40 ± 9.54	10.32%

### Comparison of ESR values among categorized levels of ESR

Correlation coefficients and Bland-Altman statistical analysis methods were utilized to assess the association and agreement within each level of ESR category. In the low ESR level category (ESR < 20 mm/h), a total of 34 patient samples were categorized. The analysis demonstrated a moderate correlation (*r* = 0.59). The paired *t*-test indicated that the MD in this ESR level category was not statistically significant, with a mean ± SD of −1.3 ± 5.0 mm/h, and LoA ranging from −11.1 to 8.5. For the medium ESR level category (ESR 20–80 mm/h), 104 samples were analyzed, revealing a very good correlation (*r* = 0.87). The paired sample *t*-test analysis revealed a non-significant MD between the two methods [MD: 0.8 ± 9.8 mm/h, *P* = 0.39], with LoA of −18.3–19.9, indicating no evidence of systematic bias. In the high ESR level category (ESR >80 mm/h), a total of 20 samples were analyzed. The comparison analysis demonstrated no significant difference between the two methods (MD = 3.2 ± 11.1 mm/h, *P* = 0.21). Moreover, there was a good correlation (*r* = 0.68) and acceptable LoA (−18.6, 25.0) between the two methods (Table 3).

## Discussion

The ESR test serves as a valuable tool in the diagnosis, management, and monitoring of various clinical conditions. Widely employed due to its simplicity and cost-effectiveness, it has become a routine test in medical settings globally (16). The Westergren method, endorsed by the ICSH, is the gold standard for ESR measurement (7). However, several confounding factors such as decreased RBC concentration in anemic patients, variations in temperature, vibration, and tube placement angle may influence ESR results obtained through this method (17). Additionally, the Westergren method posing potential risks to medical staff due to increased contact with blood specimens and heightened exposure to blood-borne infections (1, 18).

Automated methods have been developed to address the limitations of manual techniques. The SFRI ESR 3000 method is an automated system that can provide ESR results more quickly than the traditional Westergren method, which is time-consuming and requires manual reading of the sedimentation rate. This can have important clinical implications in settings where rapid decision-making is necessary, such as in emergency departments or critical care units. Furthermore, the SFRI ESR 3000 method requires a smaller blood sample volume compared to the Westergren method, requires a relatively large blood volume. This can be particularly beneficial when dealing with pediatric or geriatric patients, or those with difficult venous access. However, it is crucial to validate the automated techniques against the established Westergren method to ensure its suitability for routine use in hospitals and clinical laboratories (19). Validation studies enable laboratory technologists to select methods that are more suitable and convenient for routine settings while ensuring comparability with the standard method (20). Moreover, establishing a framework of recommendations allows clinicians and laboratory leadership to objectively assess whether and how a particular alternative ESR method can meet the clinical needs of their constituents (13).

In our study, we observed a slight increase in the ESR values obtained from the reference Westergren method compared to the automated method, although this difference was not statistically significant [MD: 0.7 ± 9.2 mm/h, *P* = 0.36]. This finding aligns closely with a study conducted in *Turkey* by Sezer et al. (6), which reported a MD of 0.19 ± 15.85 mm/h between the Westergren and automated methods (P = 0.905). Similarly, a study in Croatia by Perovic et al. (21) found no significant mean difference between the Ves-Matic Cube 200 and Westergren method [MD: 0.47 mm/h, 95% CI: 0.37 to 1.32; *P* = 0.27]. In contrast, a study in Pakistan comparing ESR values obtained by an automated ESR analyzer based on vision principles against the conventional manual Westergren method revealed a significant difference between the two methods, with a mean difference of 13.542 ± 3.041 (*P* < 0.001) (22).

The correlation analysis conducted to evaluate the association between the two ESR determination methods revealed a strong correlation between ESR results obtained using the SFRI ESR 3000 automated method and the ICSH recommended method (*r* = 0.94). This finding is consistent with previous studies conducted in Pakistan (23), Iran (24), Pakistan (10), and Croatia (21), which also reported significant strong correlations between the Westergren and different automated methods (*r* = 0.97, *p* = 0.00; *r* = 0.987, *p* < 0.001; *r* = 0.945, *p* < 0.001; *r* = 0.946, *p* < 0.001, respectively). Additionally, two studies in *Turkey by* Bogdaycioglu et al. (25) and Sezer et al. (6) reported a moderate correlation for the Ves-Matic Cube 200 method compared with the Westergren method, with correlation coefficients of 0.84 and 0.82, respectively. However, a study conducted in Iran observed a marked discrepancy in readings between the reference and automated methods (26).

The linear regression analysis performed according to Passing–Bablok to predict ESR values for the automated method from the reference Westergren method yielded the equation “y = 1.0x – 1.6.” This finding aligns with various other studies. For instance, a study conducted in Finland, comparing ESR measurements with the StaR Rsed Auto-Compact instrument to the ICSH standardized Westergren method, showed a linear regression equation between the methods as “y = 1.066x – 0.24” (27). Similarly, a study in Turkey by Sezer et al. (6) reported a regression equation of “y = 1.15x – 2.59” between the Ves-Matic Cube 200 and reference methods. In Croatia, the comparison study reported a regression equation of “y = 1.0435x – 0.0435” (*p* = 0.95) between the Ves-Matic Cube 200 analyzer and the Westergren method (21). Additionally, another study in Turkey documented a regression equation of “y = 0.92x + 1.25” between Ves-Matic Cube 200 and Westergren methods (25).

The Bland–Altman data plot revealed no evidence of systematic bias, indicating good agreement of ESR values between the Westergren and SFIR automated methods. Approximately 95% of all samples fell within the LoA of −17.3 to 18.7. The result is nearly the same as the study conducted in Turkey, which reported no evidence of systemic bias between the two methods, with a bias of −0.7 mm/h and LoA ranging from −32.6 to 31.2 mm/h (6). Similarly, a study in Pakistan showed good agreement with a bias of 2.1 mm/h between the tested analytical methods (20). Additionally, a study in Turkey documented a mean bias of 1.4 with 95% LoA of −34.4 to 37.2 mm/h for ESR measurements between Ves-Matic Cube 200 and Westergren methods (25). If this difference does not affect the interpretation, it could be possible to use both measurements. However, it is important to note that the calibration of ESR is crucial for accurate measurements due to differences in measuring principles, blood sample quality (citrate or EDTA, sampling tubes), and measuring times (27).

The imprecision was evaluated across various ESR values categorized as low (ESR < 20 mm/h), medium (ESR 20– 80 mm/h), and high (ESR >80 mm/h). For the automated method, the within-run imprecisions were found to be 27.08, 12.65, and 10.32% for low, medium, and high ESR levels, respectively. According to the CLSI H2-A4 guideline, acceptable performance limits vary for different ESR concentrations, with calculated CVs% ranging between 10.88 and 38.88 for different ESR values (28). Replicate numerical measurements can often be described by a normal, or Gaussian, distribution. In general, CV of < 10% is often considered indicative of a Gaussian distribution of replicate data, identifying a probable sufficient sensitivity of method. In our study, we observed an acceptable variations forall low, medium and high ESR levels measured by both the automated and manual ESR determination method. Similarly, a study in Turkey reported increased variations for low ESR values, with within-run imprecisions of 19.9, 10.1, and 9.90% for low, medium, and high levels, respectively, using the Ves-Matic Cube 200 system (25). Another study in Turkey also found increased imprecision at medium and low rates of sedimentation, with CVs of 14.01, 14.99, and 5.69% for low, medium, and high ESR levels, respectively, with the same system (6). Similarly, a study in Croatia reported increased imprecision at medium and low rates of sedimentation, with CVs of 9.19, 13.88, and 5.66% for low, medium, and high levels, respectively, using the Ves-MaticCube 200 analyzers (21).

### Strengths and limitations of the study

The study addresses an important aspect of clinical hematology by comparing traditional and automated methods for ESR measurement. The large sample size and the comprehensive statistical analysis (including paired *t*-tests, Pearson correlation, linear regression, and Bland-Altman plots) enhance the robustness of the findings.

The main limitation of this study is that it is a single-center study, which limits the generalizability of the findings to other populations or settings. There will also be a potential biases introduced due to the limited timeframe of data collection. Ten replicate measurements were done for each of the three samples, which represent low, medium, and high ESR levels, due to the large sample volume demand of the ESR test. However, 20 replicate measurements are recommended to determine CV% for within-run imprecision evaluation.

Though 20 replicate measurements are recommended to calculate CV% for within-run imprecision study, we only had 10 replicate measurements for each of the three samples representing low, medium, and high ESR level due to the high sample volume demand of ESR test. Another drawback of this study is that the study did not account for potential variations in ESR measurements due to patient specific factors such as underlying health conditions or medication use.

## Conclusions and recommendations

In conclusion, our study found no statistically significant difference between the manual reference Westergren method and the SFRI ESR 300 automated method for ESR determination. The current study has shown that the SFRI ESR 3000 method has demonstrated good agreement with the Westergren method, indicating that it can be a suitable alternative for ESR measurement with comparable accuracy and precision. The lack of significant bias between the two methods, as indicated by the Bland-Altman plots, supports the potential interchangeability of these methods. Interpretation using the same reference range can also be applied across both methods. However, it is important to note that the manual Westergren method remains the gold standard procedure for ESR estimation.

Implementing the SFRI ESR 3000 method in clinical practice, especially in settings with high workloads, can potentially have several impacts including, speed, workflow integration, reduction of manual errors, increased throughput, and patient satisfaction. The automated SFRI ESR 3000 method can streamline the testing process and reduce the need for manual intervention. It can minimize the potential for human errors, ensuring consistent and accurate results, even under high workload conditions. Moreover, the use of SFRI ESR 3000 method has rapid testing capabilities which may allow laboratories to handle a higher volume of ESR tests within a given time frame, helping to manage high workloads more effectively. This can significantly reduce turnaround time compared to the manual methods. The faster test results can lead to quicker diagnosis and patient management, potentially improving patient satisfaction by reducing wait times for ESR test results. Therefore, the automated SFRI ESR 3000 method of ESR measurement proves to be a reliable and suitable alternative, particularly in laboratories with high workloads. It can safely replace the Westergren method, offering efficiency and accuracy in ESR determination.

## Data availability statement

The original contributions presented in the study are included in the article/supplementary material, further inquiries can be directed to the corresponding author.

## Ethics statement

The studies involving humans were approved by Institutional Review Board of College of Medicine and Health Science, Jigjiga University. The studies were conducted in accordance with the local legislation and institutional requirements. The participants provided their written informed consent to participate in this study.

## Author contributions

MW: Conceptualization, Data curation, Formal analysis, Funding acquisition, Investigation, Methodology, Project administration, Software, Supervision, Visualization, Writing – original draft, Writing – review & editing. EA: Methodology, Software, Validation, Visualization, Writing – review & editing. AT: Data curation, Methodology, Writing – review & editing. MA: Methodology, Software, Writing – review & editing. HA: Validation, Visualization, Writing – review & editing. MMA: Data curation, Formal analysis, Supervision, Writing – review & editing. FG: Formal analysis, Methodology, Resources, Supervision, Writing – original draft.
